# Evaluating the *in vivo* effects of Aspirin on vaginal inflammation and barrier function

**DOI:** 10.3389/fimmu.2026.1901550

**Published:** 2026-09-21

**Authors:** Morgan K. Taverner, Marina Costa Fujishima, Deesha Nayar, Camila Oda, Calvin Wong, Lyle R. McKinnon, Emmanuel A. Ho, Keith R. Fowke, Thomas T. Murooka

**Affiliations:** 1 Department of Medical Microbiology and Infectious Diseases, Faculty of Health SciencesUniversity of Manitoba, Winnipeg, MB, Canada; 2 Department of Immunology, Faculty of Health Sciences, University of Manitoba, Winnipeg, MB, Canada; 3 School of Pharmacy, Faculty of Science, University of Waterloo, Kitchener, ON, Canada; 4 Centre for the AIDS Programme of Research in South African (CAPRISA), Durban, South Africa; 5 Department of Medical Microbiology and Immunology, University of Nairobi, Nairobi, Kenya; 6 Partners for Health and Development in African, Nairobi, Kenya

**Keywords:** female genital tract, mucosal inflammation, neutrophils, T cell activation, vaginal epithelial barrier

## Abstract

Inflammation is an important component of protective immunity in the lower female genital tract but must be tightly regulated to limit host tissue damage. In this study, we evaluated the *in vivo* potential of the anti-inflammatory drug Acetylsalicylic Acid (ASA) to lower proinflammatory cytokine production, restore vaginal barrier integrity and reduce the frequency of activated T cells that serve as targets for HIV infection. Daily intraperitoneal administration of ASA in mice had no effect on epithelial layer structure and CD4^+^ T cell activation status at homeostasis but significantly reduced barrier disruption in response to inflammatory bacteria *Mobiluncus mulieris (M. muleiris)*. ASA treatment lowered the frequency of activated CXCR6^+^ T_H_17 subsets in the vaginal mucosa in response to *M. muleiris* and *Gardnerella vaginalis* challenge, also reducing the number of CCR5^+^ T cells in the latter. RNAseq analysis of the draining lymph nodes indicate that ASA treatment modulates immunologic and metabolic pathways associated with inflammation, cytokine signaling and cell-cell junction proteins. Our data indicate that ASA administration may reduce inflammation and help restore vaginal homeostasis in mice and aligns with human cohort studies evaluating the potential for oral ASA to reduce HIV transmission.

## 1 Introduction

Adolescent girls and young women within the 15–24-year age group remain at a >2 fold increased risk of acquiring HIV infection compared to their male counterparts, thus preventing HIV infections among young women remains a high priority. The vaginal epithelial lining and underlying host immunity provide a robust physical and immunological barrier to prevent HIV from establishing focal infections. However, it is well-recognized that high levels of vaginal inflammation, defined by elevated pro-inflammatory cytokine expression in cervicovaginal secretions, is a strong predictor of HIV acquisition (1–3). Several factors such as ethnicity, sexual behaviors and underlying sexually transmitted infections (STIs) can independently impact the inflammatory status of the female genital tract (FGT), but the presence of bacterial communities that co-exist in the vaginal lumen, or the vaginal microbiome, has garnered significant attention as an important modulator of homeostasis and inflammation (4, 5). Women with highly diverse microbiome containing anaerobes *Gardnerella vaginalis (G. vaginalis)*, *Prevotella* spp., *Mobiluncus* spp., among others, have elevated vaginal inflammation markers (4, 6, 7), increased risk of pre-term birth (8), and higher rates of HIV infection (4, 9) compared to individuals with an optimal, *Lactobacillus crispatus (L. crispatus)* dominant microbiome. Elevated HIV acquisition risk associates with a high proinflammatory cytokine and chemokine expression profile that include IL-1β, TNF, IL-8, IL-17 and CXCL10, which are also linked to elevated numbers of activated CD4^+^ T cells in the vaginal mucosa (4, 10, 11). Importantly, a higher proinflammatory cytokine profile in the vaginal lavage fluid was associated with low HIV Pre-exposure Prophylaxis (PrEP) efficacy in the CAPRISA004 1% tenofovir gel trial, indicating that underlying inflammation can undermine an otherwise effective HIV prevention strategy in women (1). The same study also reported that high genital inflammation significantly increased HIV acquisition risk in this cohort (12). Elevated inflammatory cytokine expression are linked to biological signatures of epithelial barrier disruption, neutrophil activation and vaginal CD4^+^ T cell influx that collectively contribute to higher HIV infection rates (13, 14) Thus, multiple environmental stressors can impact vaginal inflammation, which in turn serves as a rheostat that modulate HIV acquisition risk and reproductive health complications. These studies underscore the importance of reducing inflammation and its sequalae to improve women’s reproductive health.

Acetylsalicylic acid (ASA), also known by the common name Aspirin, is a non-steroidal anti-inflammatory drug (NSAID) that has potent anti-inflammatory, analgesic and antipyretic properties. ASA exerts its effects through irreversible inhibition of the cyclooxygenase (COX) enzyme to reduce the formation of prostaglandins responsible for inducing inflammation, swelling, pain and fever (15, 16). In a study of 37 HIV uninfected women in the IQQ trial, low dose daily ASA (81mg) over a 6-week period resulted in a 35% and 28% decrease in the proportion of vaginal T cells that were CD4^+^CCR5^+^ and CCR5^+^ T_H_17 cells, respectively (17, 18). Proteome analysis showed that ASA increased vaginal barrier protein expression and reduced neutrophil degranulation markers, particularly in women with a *Lactobacillus-*dominant vaginal microbiome. These data underscore the potential for ASA to reduce the number of HIV target T cells and improve vaginal epithelial integrity as part of a safe, accessible and non-stigmatizing HIV prevention strategy for high-risk individuals.

In this study, we sought to further define the anti-inflammatory properties of ASA administration in a murine model of bacteria-induced vaginal inflammation (19). Mice given intraperitoneal administration of 32 mg/kg ASA was well tolerated for up to 6 weeks with no impact on the vaginal epithelial lining, neutrophil frequencies and CD4^+^CCR5^+^ T cell numbers and activation status at baseline. When vaginal inflammation was induced in response to *Mobiluncus mulieris (M. mulieris)* or *G. vaginalis* challenge (19), ASA pre-treatment for 7 days significantly reduced neutrophil influx into the vagina, improved barrier function and reduced the frequency of activated T cells in the lower FGT and draining lymph nodes. Specifically, pathogenic CXCR6^+^IL-17^+^ T cell frequencies were reduced by ASA treatment after challenge with anaerobes at the day 14 timepoint. Bulk RNAseq analysis of the FGT-draining lymph nodes showed that 14 day ASA treatment reduced expression of neutrophil-associated markers and modulated several immunologic and metabolic pathways associated with the inflammatory process. These studies are in alignment with data from the IQQ ASA trial and may represent an additional choice for women to reduce HIV transmission risk.

## 2 Materials and methods

### 2.1 Animals

8–10 week-old female BALB/c or C57Bl/6 mice from Jackson Laboratory were housed at the University of Manitoba Central Animal Care Services (CACS) under approved protocol 20-061. Mice were administered Aspirin daily at a dosage of 8 mg/kg (designated as “low dose”, equivalent to 39 mg daily ASA in humans) or 32 mg/kg (designated as “high dose”, equivalent to 156 mg ASA in humans) via intraperitoneal (IP) injection for up to 6 weeks. Conversion of mouse to human drug dosing was calculated using the body surface area normalization: mouse dose = human dose * human Km/mouse Km where human Km = 37 kg/m^2^ and mouse Km = 3 kg/m^2^ based on a previous study (20). For reference, daily 81 mg oral ASA treatment in humans was demonstrated to impact the frequency of HIV target cells in the lower female genital tract (18). Mice were euthanized under anesthesia by cervical dislocation prior to tissue necropsy for downstream analysis. Daily oral ASA gavage resulted in significant body weight and gastrointestinal bleeding, precluding further analysis of this experimental group.

### 2.2 ASA and salicylic acid (SA) quantitation from plasma and CVL

Collected plasma and CVL samples were stored at -80 °C prior to analysis, as described previously (19). Briefly, analysis of ASA and its metabolite salicylic acid content was performed using the Waters Acquity ultra performance liquid chromatography (UPLC) H-Class Plus system and the HSS C18 columns (100Å, 1.8 μm, 2.1 mm x 100 mm; pore size, diameter, dimensions). Mobile phases were as follows: phase A – acetonitrile (ACN) (100%); phase B – 10% acetonitrile, 90% 10 mM o-phosphoric acid aqueous buffer. For sample analysis, flow parameters of each run were kept at constant flow rate of 0.35 mL/min, with constant mobile phase composition of 22% mobile phase A and the remaining percent mobile phase B. Gradient of increasing organic phase percentage (mobile phase A) after elution of relevant analytes was sometimes used to ensure rapid clearance of potential impurities. PDA UV-Vis absorbance detector was set at 228 nm during the preparation. Plasma samples were thawed slowly in an ice-water bath. 40 µL plasma, 40 µL IS, 50 µL ACN and roughly 20 mg NaCl were added to a centrifuge tube. This mixture was vortexed for at least 5 seconds, then left to sit for at least 10 minutes. The samples were then centrifuged at 10,000 x g for 5 minutes at 4°C. The organic layer was carefully transferred to UPLC sampler vials and diluted with equal part o-phosphoric acid (10 mM). ASA and SA standard curve samples were similarly prepared, but blank plasma was spiked with ASA and SA to a final concentration in plasma of 150–6000 ng/mL.

### 2.3 Flow cytometry analysis

Female genital tract, draining lymph nodes and spleen tissues were collected, manually dissociated using surgical scissors and incubated with collagenase D (11088866001; Roche Diagnostics) for 1 hour at 37°C. Tissues were filtered through a 40μm nylon mesh strainer and single-cell suspensions washed by centrifugation and resuspended in FACS buffer (PBS + 2% FBS). Cells were stained with TruStain FcX™ (Biolegend, #101320) for 30 minutes prior to cell surface and intracellular staining with the indicated antibodies: anti-CD3 (Biolegend #100234), anti-CD4 (Biolegend #100414), anti-CD69 (Biolegend #104512), anti-CD25 (Biolegend #101910), anti-CD103 (Biolegend #121430), anti-CCR5 (Biolegend #107006), anti-CXCR6 (Biolegend #151108), anti-IFNγ (Biolegend #505840), anti-IL17A (Biolegend #506918), anti-CD69 (Biolegend #104506). Zombie Violet Fixable Viability dye was used to gate for live cells (Biolegend #423114). For cytokine expression analysis, cells were stimulated with PMA/ionomycin for 4 hours in the presence of Brefeldin A prior to fixation and permeabilization. Samples were analyzed on the BD Fortessa instrument and analyzed using FlowJo.

### 2.4 Intravaginal bacterial challenge studies

Female mice were dosed daily with either PBS or ASA at 32 mg/kg IP for 7 days, then administered 3mg 17β-estradiol subcutaneously to synchronize experimental mice to the estrous phase, three days prior to the first bacterial challenge. *L. crispatus* type strain DSM 20584, *G. vaginalis* type strain ATCC 14018 and *M. mulieris* type strain DSM 25311 were grown as previously described (19). Tubes of bacterial cultures were grown under anaerobic condition using BD GasPak Anaerobic Sachets (BD) and aliquots taken for estimation of cell counts via optical density readings (OD600). The relationship between optical density and bacterial cell count was determined using a previously determined standard curve. Mice were anesthetized with isoflurane and intravaginally challenged with 1x10^8^ cfu bacteria at days 0, 4 and 8. Cervicovaginal lavage (CVL) sampling was performed before and during bacterial challenge studies by gently washing the vaginal canal with 70 µL sterile PBS. Some of the CVL was used to assess the estrous state using Trypan Blue (Gibco™ Trypan Blue Solution, 0.4% 15250061), as described (21).

### 2.5 *In vivo* FGT integrity assay

Mice were anaesthetized with ketamine (100mg/kg) and xylazine (10mg/kg) prior to intravaginal inoculation with 50µL of 5 mg/mL lucifer yellow solution in PBS (Invitrogen™ Lucifer Yellow CH, Lithium Salt; Cat #L453) (19). After 45 minutes, mice were euthanized and the lower FGT excised and fixed in 4% (w/v) paraformaldehyde solution and frozen in OCT media for downstream microscopy analysis using the Zeiss Observer Apotome microscope and analyzed using the ZEN3 Imaging Software (Oberkochen, Germany).

### 2.6 Image analysis

Vaginal barrier permeability was quantified by measuring the mean fluorescence intensity of lucifer yellow (LY) dye detected within the epithelium or the lamina propria (using DAPI staining to delineate the two regions) using ImageJ and is defined as % LY fluorescence over a region of interest. Neutrophil recruitment into vaginal tissues was also analyzed in a similar manner using ImageJ, by calculating %Ly6G positive signal that occupy the epithelium or lamina propria defined by DAPI staining.

### 2.7 qPCR analysis

qPCR was performed using Power SYBR^®^ Green PCR Master Mix (2X) (Qiagen Cat# 4367659) and QIAGEN QuantiTect primers for murine IL-17A, IL-21, IFNγ and IL-23R by following manufacturer’s protocols. Reactions were run on the Roche LightCycler^®^ 96 and data analyzed in Excel using the housekeeping genes β-actin or HPRT to calculate 2^-ΔΔCt^ values.

### 2.8 Tissue immunohistochemistry and microscopy analysis

Tissues fixed in 4% (w/v) paraformaldehyde solution were placed in increasing sucrose solutions (10%, 20%, 30% in PBS) then embedded in OCT medium for storage at -80°C. Frozen tissues were cut into 10μm sections and mounted onto glass slides, then washed with PBS/Triton X-100 buffer (Cat #T8787, Sigma-Aldrich) twice for 5 minutes. Tissue sections were pretreated with 50ul Fc Receptor Blocked (Cat #NB309-5S, Innovex Biosciences Inc) for 30 minutes at room temperature and subsequently blocked with 2% (w/v) bovine serum, mouse serum (SP-002-VX5, ImmunoReagents Inc) and donkey serum (SP-072-VX10, ImmunoReagents Inc) for 1 hour at room temperature. Primary antibodies were diluted in blocking buffer and diluted 1:100 for anti-Ly6G (Cat #ab25377, Abcam) and 10ug/mL for anti-Human/Mouse E-cadherin (Cat#AF748, R&D Systems) for overnight staining at 4°C. Tissues were washed three times with PBS/Triton X-100 solution and incubated with secondary antibodies 1:100 Alexa Fluor 488-conjugated mouse anti-rat IgG2b (Cat# 172334 Abcam) and 1:200 Alexa Fluor 568-conjugated donkey anti-goat IgG H&L (Cat#Q22079, Thermo Fisher 1:200) for 1h at room temperature. Nuclear staining was performed using Hoechst 33342 (Cat#H1399, Thermo Fisher Scientific) and mounted with ProLong Gold antifade media (Cat#P36934, Thermo Fisher Scientific). Vaginal tissue sections were imaged using the Cytation 10 (Cytek) with further analysis performed using ImageJ.

### 2.9 Bulk RNA sequencing

FGT draining lymph nodes were collected at the indicated times, placed in RNAlater solution and RNA extracted. The raw data underwent a series of quality checks including examination of sequencing error rate, GC content distribution, and data filtering (Novogene). Sequencing error rates were all below 1% and found to be within acceptable limits, indicating high quality sequencing runs. Clean reads were then obtained by filtering the raw data by removing fragments containing sequencing adapters (adapter contamination) and reads where more than 10% of sequenced nucleotides were uncertain (N>10%). Alignment was performed and over 93% of reads for all groups were mapped to exons, followed by normalization of gene expression levels by accounting for differences in gene length and sequencing depth.

### 2.10 Bioinformatics analysis pipeline

Bioinformatics analysis was performed by Novogene. Graph visualization was performed by our laboratory using R (v4.4.1) in RStudio. *Data Quality Control:* Raw sequencing reads in FASTQ format were processed using Novogene in-house Perl scripts. Quality control removed reads containing adapter sequences, reads containing poly-N stretches, and low-quality reads from the raw data. Simultaneously, Q20, Q30, and GC content of the clean data were calculated. All downstream analyses were performed using clean, high-quality reads. *Read Mapping to the Reference Genome:* HISAT2 (v2.0.5) was used to build a genome index and align paired-end clean reads to the mm10 reference genome. *Quantification of Gene Expression Levels:* Read counts mapped to each gene were quantified using featureCounts (22) (v1.5.0-p3). FPKM (Fragments Per Kilobase of transcript sequence per Million mapped fragments) values were then calculated for each gene based on gene length and the corresponding read counts. *Principal Component Analysis:* Principal component analysis (PCA) was performed in R (v4.4.1) on the gene expression value (FPKM) of all samples. FPKM values were log2-transformed (log2[FPKM + 1]) before analysis, and PCA was conducted using the prcomp function with mean-centering applied. Sample scores for the first two principal components were plotted using ggplot2 (v4.0.2). *Differential Expression Analysis:* Differential expression analysis between two conditions/groups was performed using the DESeq2 (23) R package (v1.20.0) (24). Resulting p-values were adjusted using the Benjamini-Hochberg procedure to control the false discovery rate. Genes with an adjusted p-value ≤ 0.05 and an absolute log2 fold change ≥ 1 were considered differentially expressed. Volcano plots were generated in R (v4.4.1) using tidyverse (v2.0.0), ggplot2 (v4.0.2), and ggrepel (v0.9.6), applying the same significance thresholds (|log2 fold change| ≥ 1, adjusted p-value < 0.05) used to define differentially expressed genes. Neutrophil-related genes of interest were labeled on the plots to highlight their differential expression between conditions. *KEGG Enrichment Analysis:* Statistical enrichment of differentially expressed genes in KEGG (Kyoto Encyclopedia of Genes and Genomes) (25) pathways was tested using the clusterProfiler R package (v3.8.1). KEGG pathways with an adjusted p-value < 0.05 were considered significantly enriched. Raw datasets and analysis for all graphs in **Figure 1** are included as part of the **Supplementary Tables** and can be accessed on Harvard Dataverse (https://doi.org/10.7910/DVN/MHKURF).

**Figure 1:**
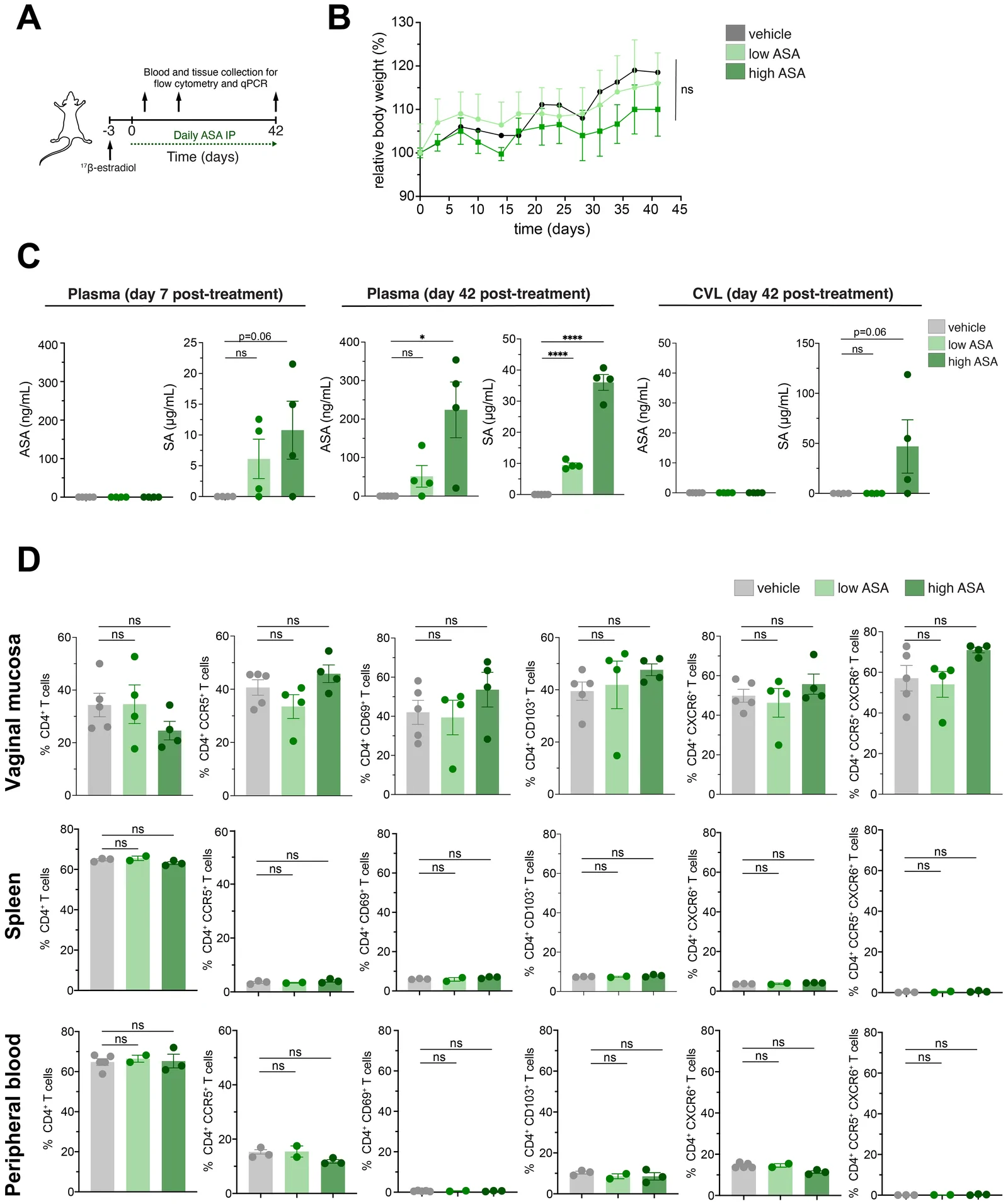
ASA administration has no impact on T cell activation status in the lower FGT at steady state. **(A)** Schematic design of the daily ASA IP treatment in female mice. **(B)** % weight change over baseline in female mice treated with vehicle, low or high ASA. ns=not significant. **(C)** ASA and SA concentrations detected in the plasma and CVL at 7 and 42 days of treatment. Each data point represents individual mice. ns=not significant, ****p <0.001. **(D)** CD4 T cell subset and phenotypic analysis in the lower FGT in the indicated groups. Representative graph from 2 independent studies shown. ns, not significant.

### 2.11 Statistical analysis

Unpaired Student’s t test and Mann-Whitney U test were used for comparisons of datasets with normal and non-normal distribution, respectively, using Prism 8 (GraphPad). One-way ANOVA was used to determine statistical differences between the means of three or more independent groups. Median and p-values from statistical analyses are indicated in each graph. When p-values were higher than 0.05, differences were considered as not significant.

## 3 Results

### 3.1 ASA administration has no impact on T cell activation status in the lower FGT at steady state *in vivo*

To examine the *in vivo* impact of ASA administration on vaginal epithelial barrier function and T cell activation within the lower FGT, female BALB/c mice were synchronized with 2mg estradiol and treated intraperitoneally with either 8mg/kg (referred to as “low”) or 32mg/kg (referred to as “high”) ASA given daily for 6 weeks (**Figure 2A**). Synchronization with estradiol normalized the influence of the estrous cycle on immune subset frequencies and activation status in the vaginal mucosa. Assessment of weight change over the course of treatment did not show significant differences between groups, albeit the high ASA dose group tended to gain weight more slowly (**Figure 2B**). ASA and its breakdown metabolite salicylic acid (SA) were detected by UPLC in the plasma at day 7 and 42 timepoint, with SA only detected in the cervicovaginal lavage in the high ASA group (**Figure 2C**). Of note, ASA is rapidly hydrolyzed in plasma (half-life in plasma is ~20 minutes) to the pharmacologically active main metabolite salicyclic acid (26). Flow cytometry analysis of FGT CD4^+^ T cells at day 42 of treatment showed no significant changes in percent CD4^+^ T cells within the CD45^+^ leukocyte gate, their activation and tissue residency markers (CD69, CD103) or activation status (CCR5, CXCR6) after the 6-week ASA treatment course (**Figure 2D**). No changes in these T cell subsets were observed in the spleen and peripheral blood. Higher CD4^+^CCR5^+^ T cell frequencies were found in the vaginal mucosa compared to peripheral blood and spleen, as expected. Taken together, daily ASA treatment at either dose induced no phenotypic changes in CD4^+^ T cell frequencies at baseline. Intraperitoneal 32 mg/kg ASA treatment was well tolerated and detectable in the plasma and cervicovaginal lavage and therefore used for all subsequent studies.

**Figure 2:**
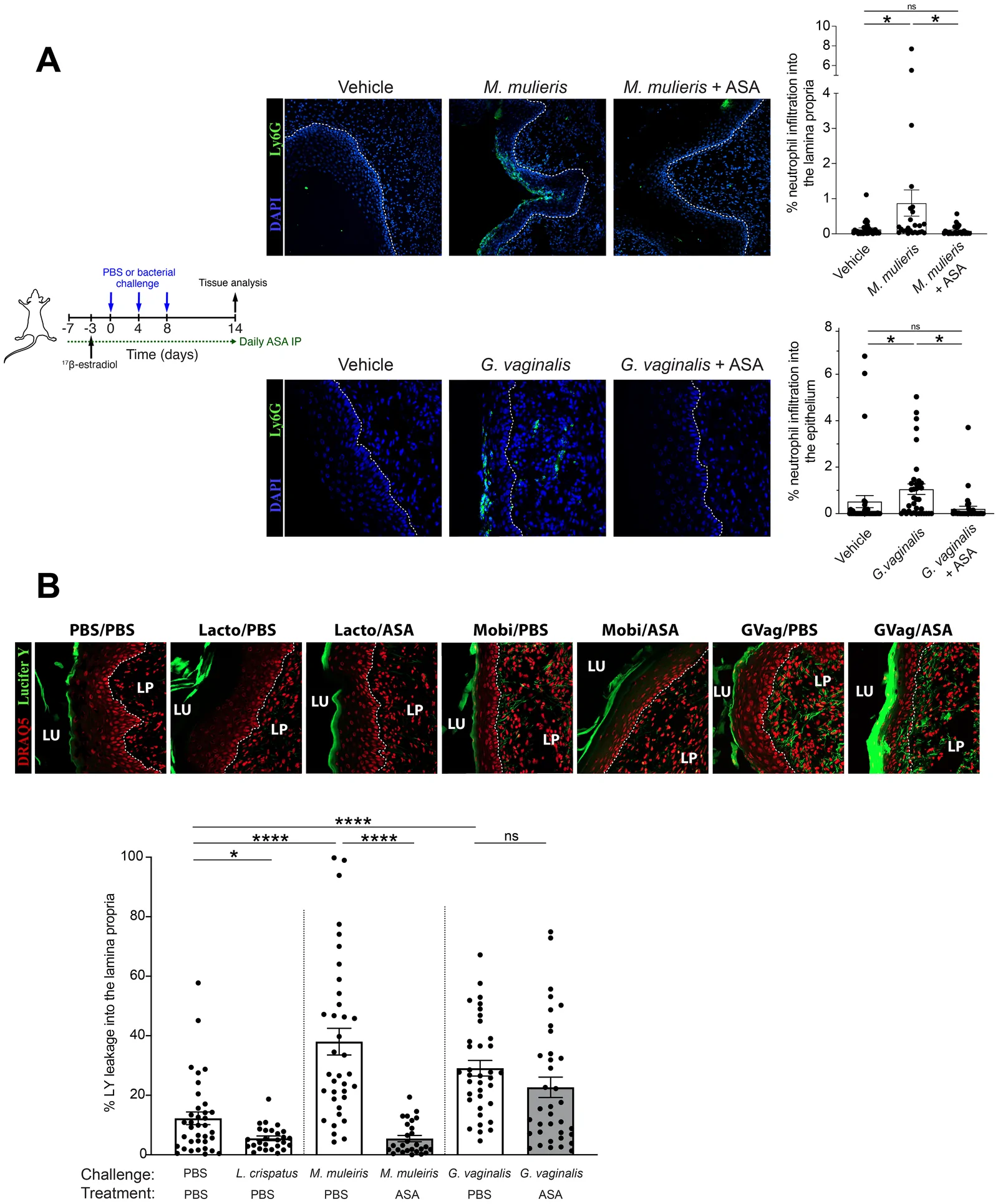
ASA administration improves vaginal barrier protection under inflammatory conditions. **(A)** Representative immunofluorescence staining of vaginal tissues at day 7 post-challenge to visualize neutrophils after bacteria challenge (representative experiment, n=4 total mice per group shown). Ly6G^+^ (green); DAPI (blue) are shown. ASA = acetylsalicylic acid. Right panel: Percentage of FGT occupied by Ly6G^+^ cells. Each data point represents quantitation from a single microscopy image after bacterial challenge, n=4 mice per group. Mean values ± SEM are shown. ns=not significant, *p <0.05. **(B)** Representative images of the vaginal tissues after bacterial challenge in the presence of absence of ASA pre-treatment. ASA = acetylsalicylic acid, Mobi = *Mobiluncus mulieris*, GVag = *Gardnerella vaginalis*. Lucifer yellow (green). Bottom panel: percentage of lamina propria occupied by lucifer yellow (LY). Each data point represents quantitation from a single microscopy image, results are from a representative study, n=3–4 total mice per group. ns, not significant, ****p <0.001.

### 3.2 ASA administration improves vaginal barrier protection under inflammatory conditions

We next evaluated the immunomodulatory potential of daily ASA IP administration in the setting of bacteria-mediated vaginal inflammation. We showed previously that intravaginal challenge with anaerobes *M. mulieris* and *G. vaginalis* initiated a neutrophil-driven inflammation that was characterized by increased neutrophil elastase (ELANE) expression, MMP8/9 secretion and significant disruption of the vaginal mucosal barrier (19). In the current study, mice were pre-treated with daily ASA 7 days prior to repeated PBS or intravaginal bacterial challenge, followed by FGT tissue collection at day 14 (**Figure 3A**). The 7-day Aspirin pre-treatment period window was chosen based on detectable SA in plasma at this time point (**Figure 2C**). IHC analysis of vaginal tissue sections confirmed neutrophil recruitment (Ly6G^+^) after *M. mulieris* challenge which was significantly reduced with ASA pre-treatment. Similarly, ASA significantly reduced neutrophil infiltration after *G. vaginalis* challenge. To measure *in vivo* vaginal barrier function, mice were placed under anesthesia and lucifer yellow dye placed intravaginally, 45 minutes prior to euthanasia and tissue collection. The presence of *M. mulieris* and *G. vaginalis* induced vaginal epithelial leakage into the lamina propria, whereas protective *L. crispatus* challenge seems to improve barrier function (**Figure 3B**). ASA treatment in the *L. crispatus* challenge group had no measurable effect on barrier function (data not shown). ASA pre-treatment significantly improved barrier function after *M. mulieris* challenge, whereas *G. vaginalis* induced barrier dysfunction was unaffected. These data indicate that ASA can reverse bacteria-induced vaginal barrier damage possibly by reducing mucosal neutrophil recruitment, albeit this affect was bacteria dependent.

**Figure 3:**
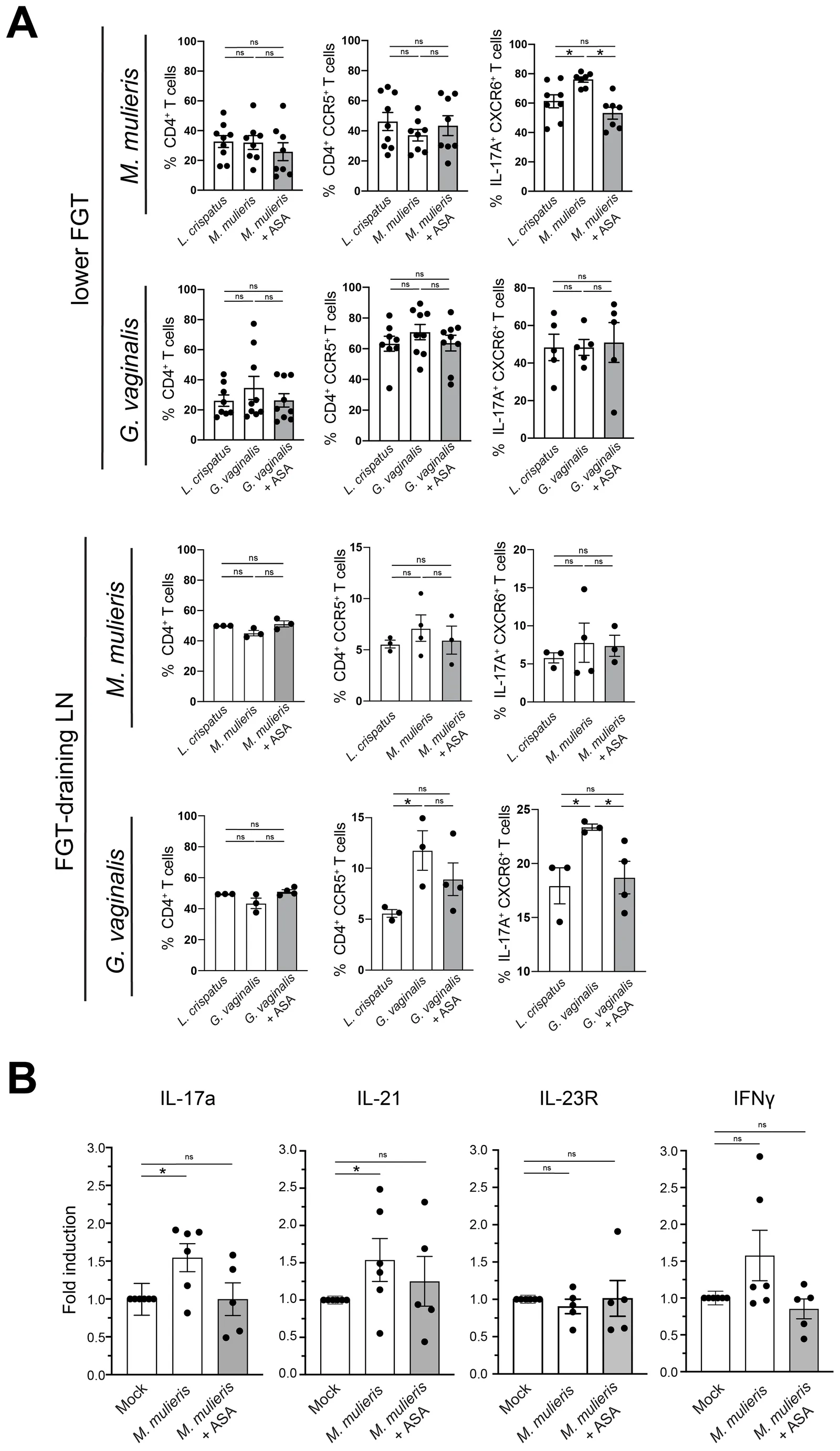
ASA administration dampens Th17 activation after intravaginal bacterial challenge. **(A)** Flow cytometry analysis of T cell subsets in the lower FGT and draining lymph node cells after challenge with the indicated bacterial species are shown. *L. crispatus* challenged mice were used as baseline comparators in each study. Representative graphs from more than 2 independent studies are shown. ns=not significant. *p<0.05. **(B)** qPCR analysis of the FGT-draining lymph node after bacterial challenge in the presence or absence of ASA pre-treatment. Data are from two independent experiments with each data point representing individual mice (n=5–6). Fold induction over mock infected controls, which were treated with PBS alone, is shown. ns, not significant. *p<0.05.

### 3.3 ASA administration dampens Th17 activation after intravaginal bacterial challenge

We next performed T cell activation and phenotypic analysis to determine whether daily ASA treatment can modulate bacteria-induced inflammatory processes. Here, cellular responses to intravaginal challenge with inflammatory anaerobes *M. mulieris* and *G. vaginalis* were compared to mice challenge with protective *L. crispatus*. Daily ASA was started 7 days prior to bacterial challenge, and the lower FGT and FGT-draining lumbar/sacral lymph nodes were harvested at 14 days post-bacterial challenge for flow cytometry and gene expression analysis by qPCR (**Figure 3A**). *M. mulieris* and *G. vaginalis* challenge did not alter CD4^+^CCR5^+^ T cell frequencies in the lower FGT, but *M. mulieris* enhanced the frequency of activated CXCR6^+^IL-17^+^ T subsets that was significantly reduced to baseline with ASA treatment. Upregulation of this activated T_H_17 population was not observed in the draining lymph node. In contrast, *G. vaginalis* increased CD4^+^CCR5^+^ T cell and CXCR6^+^IL-17^+^ T cell numbers in the draining lymph node which was significantly reduced by ASA treatment. qPCR analysis of lymph node cells confirmed upregulation of Th17-related markers IL-17A and IL-21 after normalization to mock controls that were reduced with ASA treatment, but no statistical changes to IL-23R and IFNγ transcripts were observed (**Figure 4B**).

**Figure 4:**
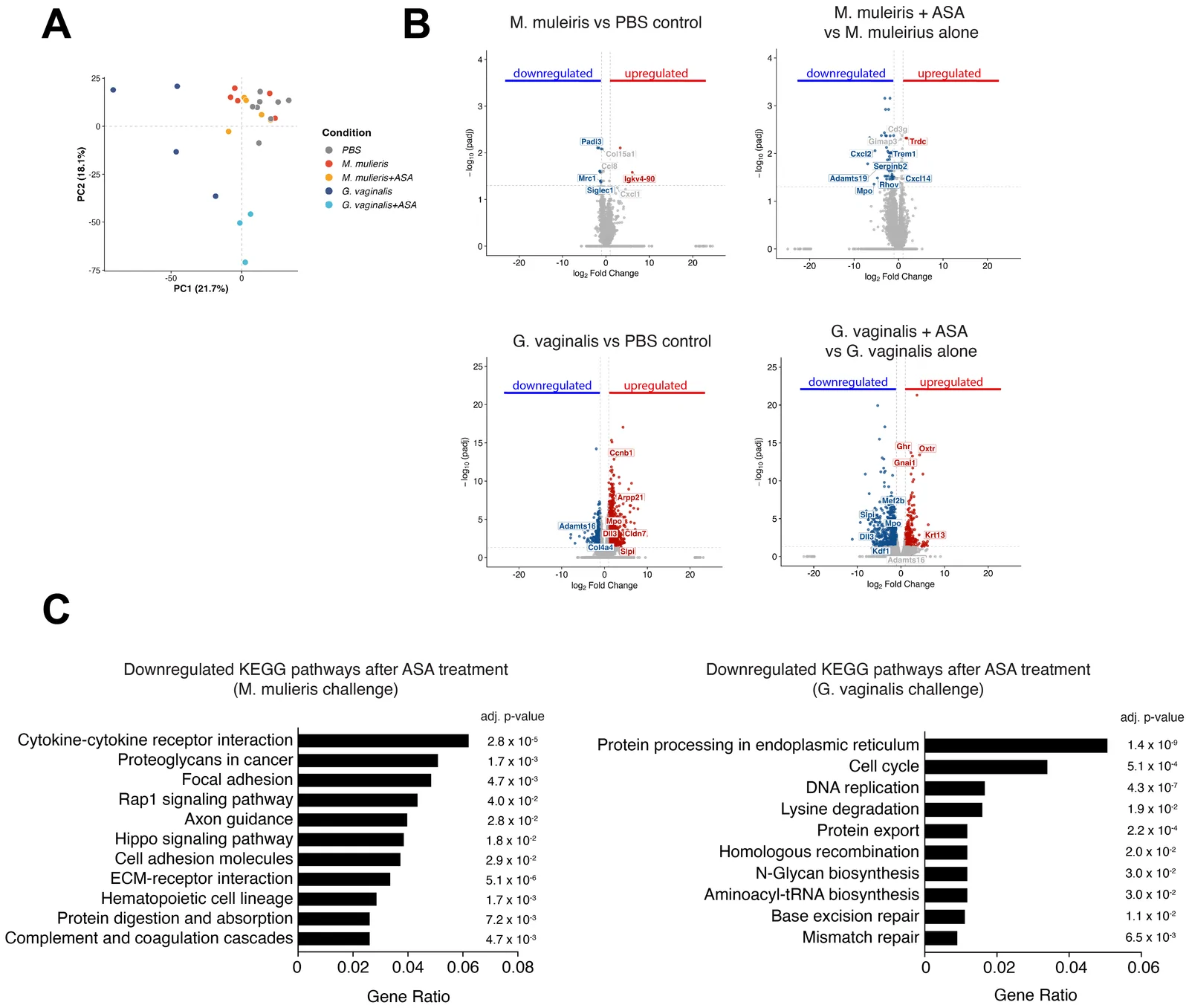
Transcriptome analysis of FGT-draining lymph node cells after bacterial challenge after ASA treatment. **(A)** Principal component analysis of the PBS group, *M. mulieris*-challenged group, *M. mulieiris* + ASA, *G. vaginalis*-challenged group and *G. vaginalis* + ASA are shown. **(B)** Volcano plot diagram of all significantly upregulated or downregulated genes in each depicted comparative analysis. Genes of interest are highlighted on the figure. **(C)** Downregulated KEGG pathway analysis after ASA treatment in *M. mulieris* and *G. vaginalis* challenged mice are shown. Adjusted p-values for each biological pathways are shown to the right. Complete datasets are found in **Supplementary Tables 1**–**6**and the Harvard Dataverse (https://doi.org/10.7910/DVN/MHKURF).

### 3.4 Transcriptome analysis of FGT-draining lymph node cells after bacterial challenge in the presence of ASA treatment

We performed bulk RNAseq analysis of the draining lymph nodes from *M. mulieris* and *G. vaginalis* challenged mice with or without ASA treatment to determine the transcriptional profile at day 14. We were not able to recover sufficient RNA from T cells isolated from the cervicovaginal tissue for sequencing analysis. PCA plot shows that mice in the PBS control (gray) and *M. mulieris* challenge groups (red) clustered together whereas *G. vaginalis* challenge (blue) resulted in distinct clustering of data points (**Figure 1A**). Interestingly, ASA treatment shifted gene expression profiles in *G. vaginalis* but not *M. mulieris* challenged mice. Vaginal challenge with *G. vaginalis*, and to a lesser extent with *M. mulieris*, resulted in upregulation of several inflammatory and neutrophil-associated genes compared to controls that were downregulated with ASA treatment in *M. mulieris* (*Cxcl2*; p=8.7x10^-2^, *Serpinb2;* p=2.9x10^-2^, *Mpo;* p=4.3x10^-2^, *Trem1;* p=1.3x10^-2^) and *G. vaginalis* (Mpo; p=2.8x10^-4^, *Slpi;* p=3.9x10^-7^) challenged mice (**Figure 1B**, **Supplementary Table 1**). KEGG analysis showed that ASA downregulated a number of pathways involved in altered lipid metabolism, cytokine signaling, expression of extracellular matrix proteins and the peroxisome proliferator-activated receptor (PPAR) signaling pathway, the latter serving as a receptor for prostaglandins and regulator of immune activation and inflammation^171^. Together, ASA treatment can modulate immunological markers in the FGT-draining lymph nodes in response to inflammatory bacteria.

## 4 Discussion

The female genital tract regulates complex reproductive processes while interfacing with an array of external stimuli, including microbial, physiological and environmental stressors that can lead to induction of an inflammatory response (27). It is well recognized that prolonged vaginal inflammation, regardless of the cause, can negatively impact women’s reproductive health including increased risk of pre-term birth (8), cervical dysplasia (28) and sexually-transmitted infections (STIs) (4, 9, 29). In this study, we evaluated whether ASA treatment can improve vaginal mucosal barrier function and reduce T cell activation in an *in vivo* model of bacteria-induced inflammation. Consistent with findings from the IQQ ASA trial, Aspirin treatment in mice reduced neutrophil influx to vaginal mucosa, improved barrier integrity in the presence of anaerobe *M. mulieris* and reduced CXCR6^+^ T_H_17 frequencies in the vagina and draining lymph nodes. RNAseq analysis of FGT-draining lymph nodes revealed that ASA can downregulate inflammatory and neutrophil-related markers known to impact vaginal barrier function. Collectively, our data suggest that ASA has the potential to be a safe, affordable and non-stigmatizing HIV prevention drug.

The stratified squamous epithelial layer of the lower female genital tract provides a multi-layered barrier to prevent pathogen invasion and coordinate immune responses^117^. The presence of *Lactobacillus* species further augments this protective phenotype through the production of lactic acid and antimicrobial compounds that limit outgrowth of pathogenic anaerobes. However, a microbial shift toward a diverse, non-*Lactobacillus* microbiome is associated with heightened inflammation and a 4-fold increase in HIV transmission in young South African women (4). The detection of pathogens by vaginal epithelial cells and subsequently activated signaling pathways are likely mediated by TLRs on the surface of vaginal epithelial cells (30, 31). Antigen presenting cells (APCs) are also able to sense and respond to BV-associated bacteria through TLR signaling pathways resulting in their activation and initiation of inflammatory responses (5). We previously showed that vaginal challenge with anaerobes *M. muleiris* and *G. vaginalis* induced robust neutrophil recruitment that led to disruption of the vaginal epithelial layer through production of matrix metalloproteases and elastase (19). We show that ASA treatment can significantly reduce neutrophil influx into the lower FGT in response to both anaerobes tested, but vaginal barrier integrity improved only in *M. muleiris* challenged mice. *G. vaginalis* produce virulence factors sialidase and vaginolysin that can degrade the vaginal mucus barrier and have cytolytic activity against epithelial cells to compromise integrity of the vaginal epithelial lining: we hypothesize that ASA does not reverse tissue damage induced by these bacterial factors (32). Similarly, changes in the gut microbiota composition can regulate neutrophil function, where signals sensed in local and distal organs modulate their accumulation, survival and magnitude of the ensuing inflammatory response (33–36). Neutrophil activation and release of neutrophil extracellular traps (NETs) are associated with severe lung pathology in COVID-19 patients where neutrophils can directly kill pneumocytes *in vitro (*37) and recently shown to trigger bone destruction during periodontitis (38). In the current study, ASA pre-treatment reduced neutrophil recruitment into the vaginal submucosa, resulting in improved barrier function after challenge with *M. mulieris* but not *G. vaginalis*. While ASA has not been shown to directly modulate epithelium function, ASA can reduce neutrophil activation and NETs release *in vitro (*39, 40). In an *in vivo* model of *Staphylococcus aureus* induced sepsis, ASA pretreatment significantly reduced platelet aggregation and release of prothrombotic NETs in the liver microvasculature (41). These studies highlight the potential for ASA to block neutrophil activation and NET release, thereby preventing tissue damage of the vaginal barrier in response to specific anaerobes.

IL-17 is a key cytokine that plays an important role in maintaining vaginal epithelial barrier integrity and is particularly important for host defense against extracellular bacterial and fungal infections (42). IL-17 is produced by Th17 cells, γδ T cells, natural killer (NK) cells, natural killer T (NKT) cells and group 3 innate lymphoid cells (ILC3) and help regulate neutrophil recruitment through induced expression of neutrophil chemokines CXCL1/2 (43, 44). However, activated T_H_17 subsets have been described to express high levels of granzymes, perforins and IFNγ and are drivers of pathology in a model of experimental autoimmune encephalomyelitis (EAE) (45, 46). Our data show that ASA pre-treatment reduced CXCR6^+^IL-17^+^ T cell frequencies in both vaginal tissue and lymph node without reducing IL-17 gene expression. CXCR6 expression, along with CD69, are enriched in the T_RM_ fractions in the cervix (47, 48) and can serve as a co-receptor for HIV infection (49). These observations indicate that ASA may reduce the number of activated T cells that are highly susceptible to HIV infection (50, 51), which has direct implications on HIV transmission across the vaginal mucosa. One possibility is that ASA-mediated improvement of the vaginal barrier integrity helps limit microbial exposure and dampen immune activation potential in this compartment. Another possible explanation is that ASA interferes with neutrophil-T_H_17 crosstalk (52–55). Neutrophils and NETs, through the release of histones, can directly activate T cells and enhance T_H_17 cell differentiation that is dependent on TLR2 (53). Neutrophils have been shown to potentiate T_H_17 cell differentiation and recruitment into the oral mucosa to mediate tissue destruction in a murine model of periodontitis (38). Sensitization with allergen and endotoxin in the lungs led to the production of enucleated neutrophil cytoplasts that promotes T_H_17 differentiation in the lung draining mediastinal lymph nodes (54). In turn, activated T_H_17 cells can produce neutrophil chemokines CXCL1/2 and CXCL8 to reciprocate neutrophil recruitment across different disease contexts (55–57). Therefore, breaking the neutrophil-T_H_17 amplification loop during vaginal inflammation may promote wound healing programs and help restore immune homeostasis. Despite its long history as an immunomodulatory and anti-inflammatory drug, Aspirin use is not associated with increased STI risk or susceptibility to other bacterial/viral infections. For example, the ANTISEPSIS sub study found similar rates of death associated with sepsis in older adults taking 100mg daily aspirin in the US and Australia (58). Thus, ASA treatment may be particularly beneficial in individuals with high vaginal inflammation to achieve genital ‘immune quiescence’ or a state of lower inflammation without compromising the ability to mount a specific and highly regulated immune response to pathogens, as observed in highly exposed but seronegative individuals (HESN) (59, 60).

This study has several limitations. Due to technical challenges in obtaining sufficient RNA from T cells in the lower FGT, transcriptome analysis was performed on FGT-draining lymph node cells. While this may provide a proxy for ASA-mediated immunological changes in the lower FGT, our analysis does not consider gene expression profiles of epithelial and mucosa-associated immune cells after direct contact with vaginal bacteria. This may explain the low number of differentially expressed genes observed in response to *M. mulieris* in our study, which contrasts with robust upregulation of inflammatory pathways observed when cervicovaginal epithelial cells were challenged with *M. mulieris in vitro* (61). It would be of interest to determine whether *M. muleiris* released extracellular vesicles (bEVs) can imprint host immune responses beyond the genital mucosal barrier in a TLR2/5 dependent manner *in vivo*. Another limitation is that PBS-treated mice was used as a comparator for our gene expression analysis rather than *Lactobacillus*-treated mice, which removes any bystander effects from *Lactobacillus crispatus* challenge alone. Improved tissue digestions protocols to perform single-cell sequencing platforms would better inform how ASA modulate bacteria-induced inflammatory pathways across different immune and stromal cell subsets.

Inflammation is an important component of protective immunity but must be tightly regulated to limit damage to host tissues. This study evaluated the potential for ASA to improve vaginal barrier function and decrease activated T cell frequencies toward the goal of reducing HIV transmission risk in women. Our data points to the ability of ASA to reduce vaginal epithelial damage by limiting neutrophil recruitment and potentially interfering with the neutrophil-T_H_17 crosstalk. These data underscore the potential for ASA as an additional choice for women to reduce HIV transmission risk that is safe, accessible and non-stigmatizing.

## Data Availability

All data needed to evaluate the conclusions in the paper are presented in the paper. RNAseq analysis datasets are uploaded on Harvard Dataverse https://doi.org/10.7910/DVN/MHKURF.
