# Correction: Evaluating the *in vivo* effects of Aspirin on vaginal inflammation and barrier function

**DOI:** 10.3389/fimmu.2026.2010437

**Published:** 2026-10-05

**Authors:** Morgan K. Taverner, Marina Costa Fujishima, Deesha Nayar, Camila Oda, Calvin Wong, Lyle R. McKinnon, Emmanuel A. Ho, Keith R. Fowke, Thomas T. Murooka

**Affiliations:** 1 Department of Medical Microbiology and Infectious Diseases, Faculty of Health SciencesUniversity of Manitoba, Winnipeg, MB, Canada; 2 Department of Immunology, Faculty of Health Sciences, University of Manitoba, Winnipeg, MB, Canada; 3 School of Pharmacy, Faculty of Science, University of Waterloo, Kitchener, ON, Canada; 4 Centre for the AIDS Programme of Research in South African (CAPRISA), Durban, South Africa; 5 Department of Medical Microbiology and Immunology, University of Nairobi, Nairobi, Kenya; 6 Partners for Health and Development in African, Nairobi, Kenya

**Keywords:** female genital tract, mucosal inflammation, neutrophils, T cell activation, vaginal epithelial barrier

There was a mistake in the caption of **Figures 1, 2, 3** and **4** as published.

The caption for **Figure 1** was incorrectly placed under **Figure 2**.

The caption for **Figure 2** was incorrectly placed in the **Figure 3**.

The caption for **Figure 3** was incorrectly placed in the **Figure 4**.

The caption for **Figure 4** was incorrectly placed in the **Figure 1**.

The original version of this article has been updated.

